# An improved YOLOv8n-IRP model for natural rubber tree tapping surface detection and tapping key point positioning

**DOI:** 10.3389/fpls.2024.1468188

**Published:** 2024-10-30

**Authors:** Xirui Zhang, Weiqiang Ma, Junxiao Liu, Ruiwu Xu, Xuanli Chen, Yongqi Liu, Zhifu Zhang

**Affiliations:** ^1^ School of Mechanical and Electrical Engineering, Hainan University, Haikou, China; ^2^ School of Information and Communication Engineering, Hainan University, Haikou, China

**Keywords:** tapping surface detection, key point positioning, intelligent rubber-tapping robot, receptive-field attention, AFPN

## Abstract

Aiming at the problem that lightweight algorithm models are difficult to accurately detect and locate tapping surfaces and tapping key points in complex rubber forest environments, this paper proposes an improved YOLOv8n-IRP model based on the YOLOv8n-Pose. First, the receptive field attention mechanism is introduced into the backbone network to enhance the feature extraction ability of the tapping surface. Secondly, the AFPN structure is used to reduce the loss and degradation of the low-level and high-level feature information. Finally, this paper designs a dual-branch key point detection head to improve the screening ability of key point features in the tapping surface. In the detection performance comparison experiment, the YOLOv8n-IRP improves the D_mAP50 and P_mAP50 by 1.4% and 2.3%, respectively, over the original model while achieving an average detection success rate of 87% in the variable illumination test, which demonstrates enhanced robustness. In the positioning performance comparison experiment, the YOLOv8n-IRP achieves an overall better localization performance than YOLOv8n-Pose and YOLOv5n-Pose, realizing an average Euclidean distance error of less than 40 pixels. In summary, YOLOv8n-IRP shows excellent detection and positioning performance, which not only provides a new method for the key point localization of the rubber-tapping robot but also provides technical support for the unmanned rubber-tapping operation of the intelligent rubber-tapping robot.

## 1 Introduction

As the only renewable industrial raw material and strategic resource, natural rubber is often categorized as one of the four major industrial raw materials, along with steel, petroleum, and coal. Due to the unique physical properties of natural rubber: resilience, elasticity, abrasion resistance, impact resistance, efficient heat dissipation, and flexibility at low temperatures that cannot be replaced by synthetic alternatives, it is widely used in more than 50,000 products, such as aircraft tires, sporting goods, medical and scientific instruments, and insulated cables, which has led to a significant increase in the annual demand for natural rubber (Tan et al., 2023). According to the statistical report of the Rubber Research Institute of the Chinese Academy of Tropical Agricultural Sciences, the global natural rubber production in 2023 is 14.319 million tons, up 0.5%. The natural rubber consumption is 15.19 million tons, an increase of 0.8%. The global natural rubber production is forecast to reach 14.542 million tons in 2024, up 1.6%. The consumption is predicted to reach 15.67 million tons, an increase of 3.0%. At present, natural rubber tapping is mainly used to tap rubber by hand, and the commonly used rubber tapping tools are traditional tapping knives, handheld electric tapping knives, etc (Arjun et al., 2016; Soumya et al., 2016; Zhou et al., 2021). A rubber-tapping worker needs to tap more than 500 rubber trees per day, which is labor-intensive and requires high skills. However, rubber trees are mainly planted in the developing countries of Asia and South America, affected by the economic situation, regional politics, environmental climate, and many other factors, resulting in the price of natural rubber never a steady increase and even some decline. A severe blow to the motivation of workers resulted in the loss of many skilled workers and large areas of rubber forests facing abandonment, so the natural rubber industry is facing a labor shortage and an aging bottleneck (Zhou et al., 2022). Therefore, there is an urgent need to develop an intelligent rubber-tapping machine to reduce the work intensity of rubber workers, increase rubber-tapping yield, and solve the predicament of the natural rubber industry (Zhou et al., 2022). Among them, using machine vision to detect the tapping area and locate the starting and ending point of tapping is the key to realizing intelligent tapping. The rubber tapping area is composed of spiral lines tapped by rubber workers. The starting and end points of rubber tapping are located at the beginning and end of the spiral line. Whether the starting and end points of rubber tapping can be accurately positioned affects the quality and yield of rubber. However, during rubber tapping operations in rubber forests, complex factors such as uneven light exposure, different thicknesses of rubber trees of various ages, and unclear tapping line features make it difficult to locate the starting and ending points of tapping accurately.

In fact, the characteristics of the key points of tapping are small features located on the tapping surface. Therefore, whether the key points of tapping can be accurately located depends on whether the detailed features of the tapping surface can be fully extracted and whether the characteristics of the key points of tapping can be screened out from the numerous detailed features. This is similar to the problems encountered in most object detection tasks in the agricultural field, namely, how to extract object features and filter out important features. In recent years, with the development of machine vision and agricultural intelligence, machine vision has been widely applied in the agricultural field (Rehman et al., 2019), including the application of traditional machine learning methods and deep learning methods. In traditional machine learning methods, the object detection task is mainly performed by manually designed classifiers using the object’s color, geometric, and texture features to classify and detect the object. For example, Tan et al. (2018) used the histogram of gradient direction and color features to distinguish blueberry fruits of different maturity. Lin et al. (2020) detected apricot varieties based on features of contour information. Li et al. (2016) combined color, shape and texture features to identify unripe green citrus fruits. The above methods have achieved certain results, but at the same time, they have also exposed some drawbacks. Traditional machine learning methods require a lot of time to perform manual feature selection and have limited adaptability in complex scenarios, which greatly hinders the performance and robustness of traditional machine learning methods for object detection in natural environments (Chen et al., 2024). This is extremely disadvantageous for detecting the tapping surface and locating the key points of rubber tapping in the complex rubber forest environment.

Deep learning is a powerful subcategory of machine learning. It can increase the depth and width of the entire large network through the continuous stacking of small modules, thereby improving the feature extraction capabilities of the network and having stronger feature extraction ability than traditional machine learning. At the same time, deep learning does not require manual feature selection and is highly adaptable to complex scenarios. Therefore, deep learning has become the preferred technology for identification and detection in the agricultural field (Liu and Liu, 2024; Altalak et al., 2022). So far, deep learning has been widely studied in many agrarian applications (Thakur et al., 2023), including weed detection (Chen et al., 2022; Ortatas et al., 2024), pest and disease detection (Kumar and Kukreja, 2022; Tang et al., 2024), fruit detection (Wang et al., 2023c; Guan et al., 2023), grain crop detection (Song et al., 2023; Wang et al., 2023b), and so on. Among them, the YOLO model, as a representative of the one-stage detection algorithm model, is slightly inferior to the two-stage detection algorithm models, such as Faster-RCNN and Mask-RCNN, in terms of detection accuracy, but its lightweight network structure design enables it to have a faster detection speed and a smaller model size. So, it has been widely used in various fields (Bello and Oladipo, 2024; Wang et al., 2023a; Mokayed et al., 2023). However, the two-stage detection algorithm model has a large number of parameters and requires greater computing power, which poses a challenge to the deployment of the model on the mobile terminal. In fact, the computing resources of the intelligent rubber-tapping robot are limited, and the detection speed will be seriously affected compared with the hardware configuration in the experimental environment. Therefore, the YOLO series model is more suitable for deployment in the rubber-tapping robot to realize intelligent rubber tapping.

At present, researchers have conducted little research on intelligent rubber tapping. Sun et al. (2022) proposed a natural rubber tree tapping trajectory detection method based on an improved YOLOv5 model, which realized the detection of the tapping surface and achieved a mAP50 of 95.1%. Chen et al. (2023) proposed a natural rubber tree tapping area detection and new tapping line positioning method based on an improved mask region convolutional neural network (Mask-RCNN), which realized the segmentation and extraction of tapping lines and located new tapping lines based on existing tapping lines, with the segmentation accuracy of tapping lines reaching 99.78%. The above scholars discussed the tapping surface and tapping line, respectively, but lacked research on the positioning of the starting and end points of tapping. Positioning the rubber-tapping starting point is the first step of the whole process. Without determining the position of the starting point of rubber tapping, the follow-up work of rubber tapping cannot be carried out. The accuracy of the positioning of the starting point of rubber tapping directly affects the quality of the glue flow after tapping. Positioning the end point of rubber tapping is the final step of the entire rubber tapping process, which involves the length of the tapping line. Currently, the commonly used secant lengths are 1/2 secant (the tapping surface is 1/2 of the rubber tree surface) and 1/4 secant (the tapping surface is 1/4 of the rubber tree surface). The efficiency of rubber flow is different for different tapping line lengths. Therefore, the accurate positioning of the starting and end points of tapping is of great significance in the whole tapping process. To this end, this paper proposes an improved YOLOv8n-IRP (Improved rubber tapping key point positioning) model based on YOLOv8n-Pose, which is used to detect the tapping surface of rubber trees and locate the starting and end points of rubber tapping. YOLOv8n-Pose is an end-to-end network that integrates object detection and key point detection. Its lightweight network structure makes it difficult for its detection and positioning accuracy in complex rubber forest environments to meet the actual rubber tapping requirements. To address this problem, this paper makes three improvements to the model. The main work and contributions are as follows:

(1) A data set of natural rubber tree tapping surface detection and starting point and end point positioning, including tapping surfaces of different tapping ages and tapping surfaces with different angles and light intensities, is established. Methods such as noise addition and picture splicing are used to preprocess the data set to improve the generalization ability and robustness of the model.
(2) The Receptive-field attention mechanism is integrated into the backbone network, which solves the problem of parameter sharing of larger convolution kernels in ordinary convolutions and calculates the importance of all features in the receptive field, thus improving the backbone network’s feature extraction capability.
(3) The Asymptotic Feature Pyramid Network (AFPN) replaces the Path Aggregation Feature Pyramid Network (PAFPN) of the neck network, reducing the loss or degradation of high-level feature information in the top-down enhancement process and the loss and degradation of low-level feature information in the bottom-up enhancement process.
(4) A dual-branch key point detection head is designed based on the residual module. The dual-branch structure uses the sigmoid function as a gate to generate different weights for the two branches to screen out important features, while the residual structure makes up for important features lost during the feature screening process, enabling important features to be screened out as completely as possible.

## 2 Materials and methods

### 2.1 Data collection and annotation

**Figure 1:**
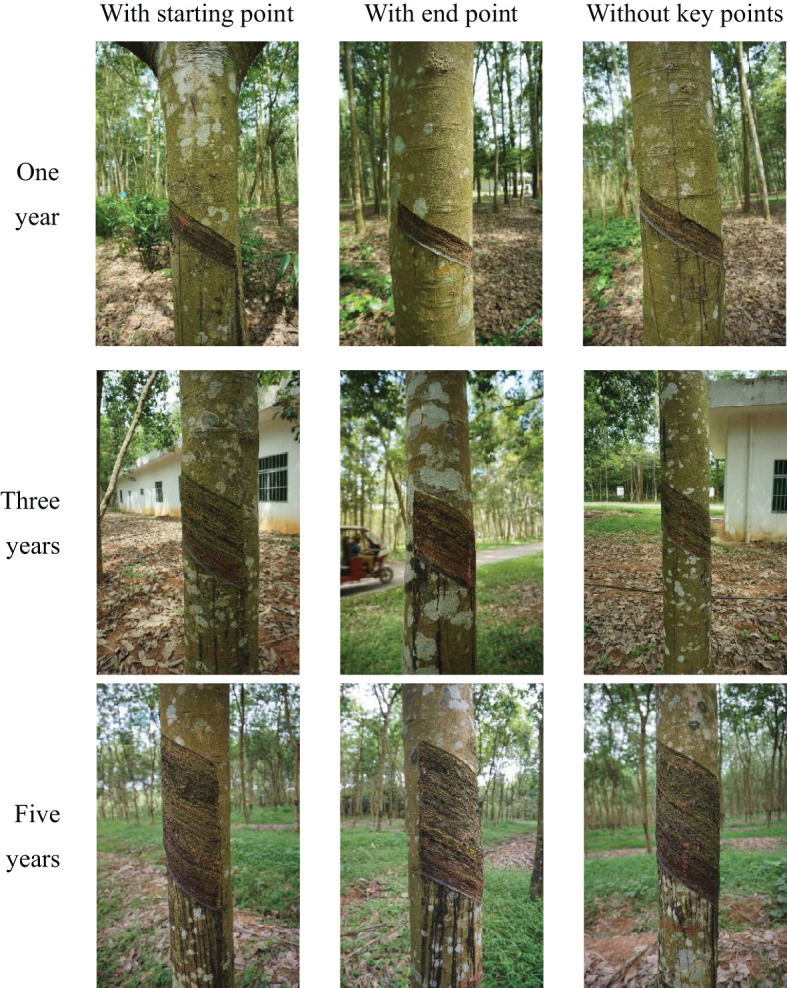
Representative sample data set of different tapping ages.

The experiment is conducted on rubber trees tapped for one, three, and five year(s). In the National Natural Rubber Forest in Danzhou City, Hainan Province, China, 2029 photos are collected using image acquisition equipment, a Sony Alpha 6000 camera with a resolution of 4000×6000. In order to ensure the richness and diversity of the samples, multi-angle shooting methods are used under different lighting conditions, and photos of 9 scenes are collected. It includes rubber trees with one, three, and five year(s) of tapping age. The rubber trees of each tapping age also include rubber trees that block the end point of tapping but not the starting point of tapping, rubber trees that block the starting point of tapping but not the end point of tapping, and rubber trees that both the starting point and end point are blocked at the same time, as shown in **Figure 1**. Finally, Labelme image annotation software is used to manually label the rubber tree’s tapping area, tapping starting point, and tapping endpoint to create a JSON format data set.

### 2.2 Data enhancement

**Figure 2:**
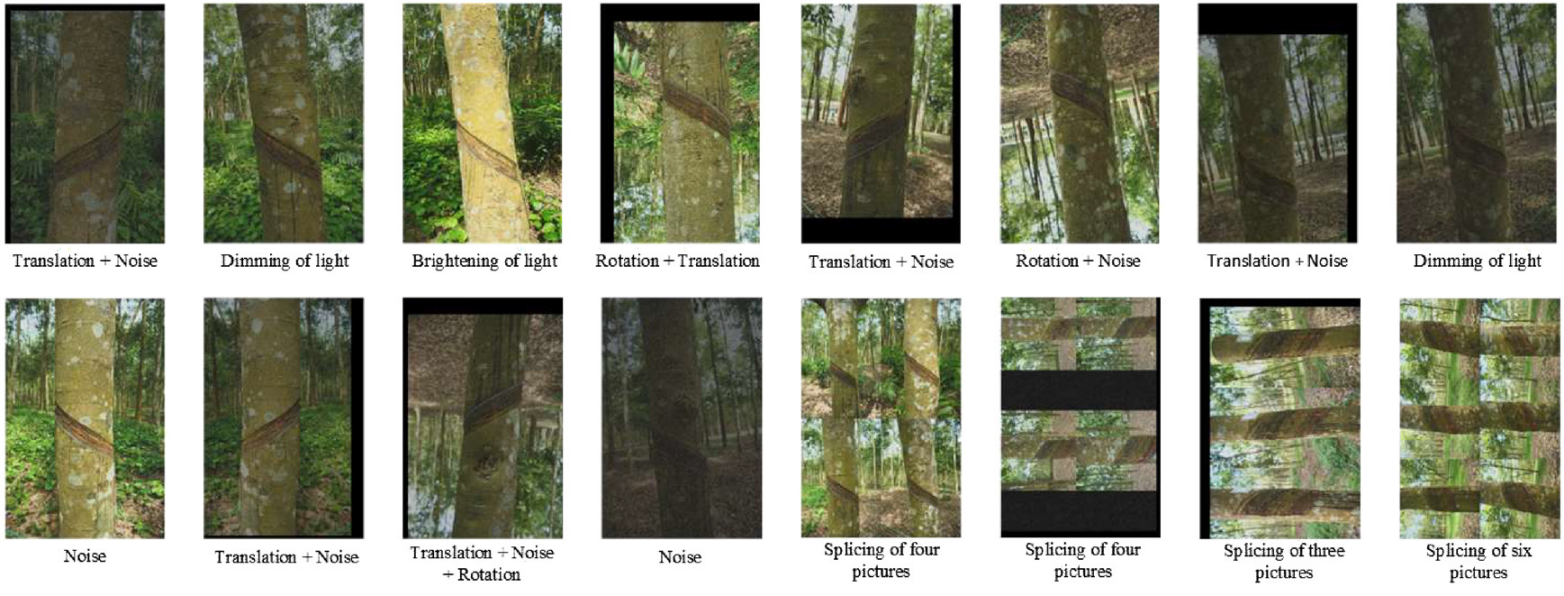
Sample data enhancement.

In deep learning network model training, the richness, diversity, and accuracy of the data set have a decisive impact on the final training results of the network model. The singleness and deficiency of the data set will lead to the model being overfitted. At the same time, due to the complex environment of the rubber forest, the use of machine vision to collect the tapping surface information of the rubber tree will be affected by unfavorable factors such as light and noise, which will lead to significant errors in the final identification and positioning. Therefore, it is necessary to enhance further the data set before network training to prevent over-fitting of the model and improve the generalization ability of the network model to adapt to the complex rubber forest environment. This study performed various random enhancement operations on the annotated original data set, including adding noise, changing light, changing pixels, translation, stitching multiple pictures, and flipping, as shown in **Figure 2**. In order to ensure the balance of the proportions of various categories in the data set, a method of different enhancement times for other categories is adopted. Categories with a smaller proportion have an increase in times of enhancement, while categories with a larger proportion have a reduced number of improvements. Finally, it is divided into a training set, a verification set, and a test set in a ratio of 8:1:1. The number of pictures is 5712, 715, and 715, respectively. **Table 1** shows the change in the number of category labels before and after the enhancement.

**Table 1:** The number of category labels before and after data augmentation.

Category	Original	Data Enhancement
starting-point	1053	2106
ending-point	244	1220
non-point	732	2196
Mixed of three categories	0	1620

### 2.3 Standard YOLOv8 network structure

YOLO (You Only Look Once) is the beginning of the One-Stage detection algorithm. Compared with Two-Stage algorithms, YOLO can greatly improve the detection speed while ensuring good detection accuracy. According to the scale of the network, the YOLOv8 model can be divided into five versions, namely YOLOv8n, YOLOv8s, YOLOv8m, YOLOv8l, and YOLOv8x, and each version includes three versions of object detection, segmentation, and key point detection. Considering the actual rubber-tapping situation, this article selected the lightweight YOLOv8n key point detection algorithm for research. Compared with the other four, YOLOv8n has a lightweight parameter structure, which is more conducive to deployment in small mobile devices.

**Figure 3:**
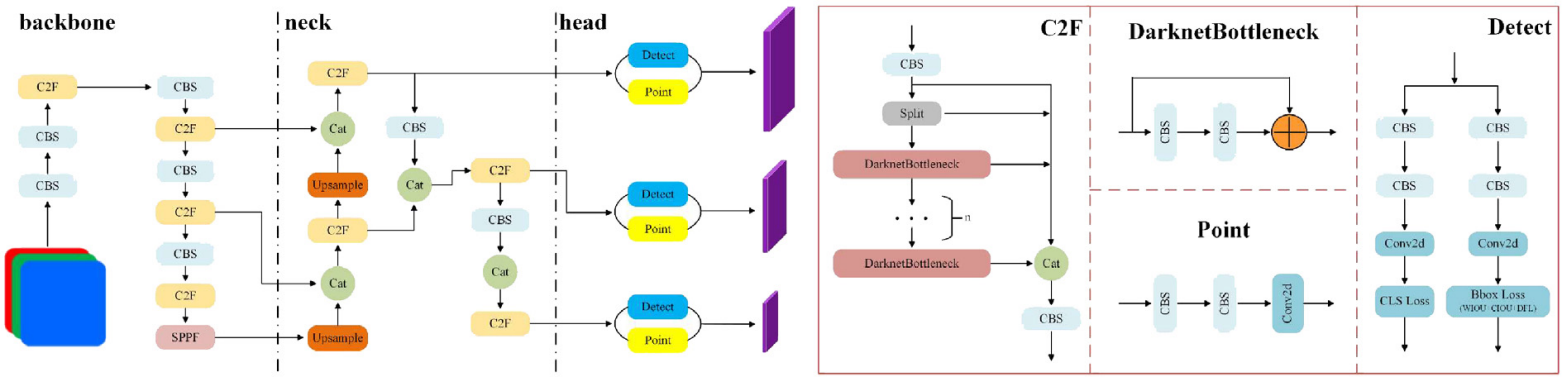
The architecture of Standard YOLOv8 Key point detection model.

The key point detection network structure of YOLOv8 is composed of a backbone network, a neck network, and a head network, as shown in **Figure 3**. First, the input image enters the backbone network within which the CBS module, C2F module, and SPPF module are used to extract features at various scales. Then, the neck network uses the Path Aggregation Feature Pyramid Network (PAFPN) structure to process further and fuse the extracted multi-scale features. Finally, the head network processes the fused feature maps at different levels to output the detection results.

## 3 Improved YOLOv8n-IRP network structure

### 3.1 Enhancement of backbone network feature extraction capabilities

Traditional convolution uses the same parameters in each receptive field to extract feature information through the convolution kernel without considering the different information between different positions. This results in a large amount of redundant information in the extracted data, which reduces the extraction time. The efficiency of features greatly limits the performance of the model. The emergence of the spatial attention mechanism enables the model to focus on certain key features (Park et al., 2020; Li et al., 2019; Luo et al., 2022), enhancing the network’s ability to capture detailed feature information. However, it can only be used to solve the identification of spatial features and does not completely solve the parameter-sharing problem of larger convolution kernels (such as 3×3 convolution). In addition, they cannot judge the importance of each feature in the receptive field, such as the existing Convolutional Block Attention Module (CBAM) (Woo et al., 2018) and Coordinate Attention(CA) (Hou et al., 2021).

**Figure 4:**
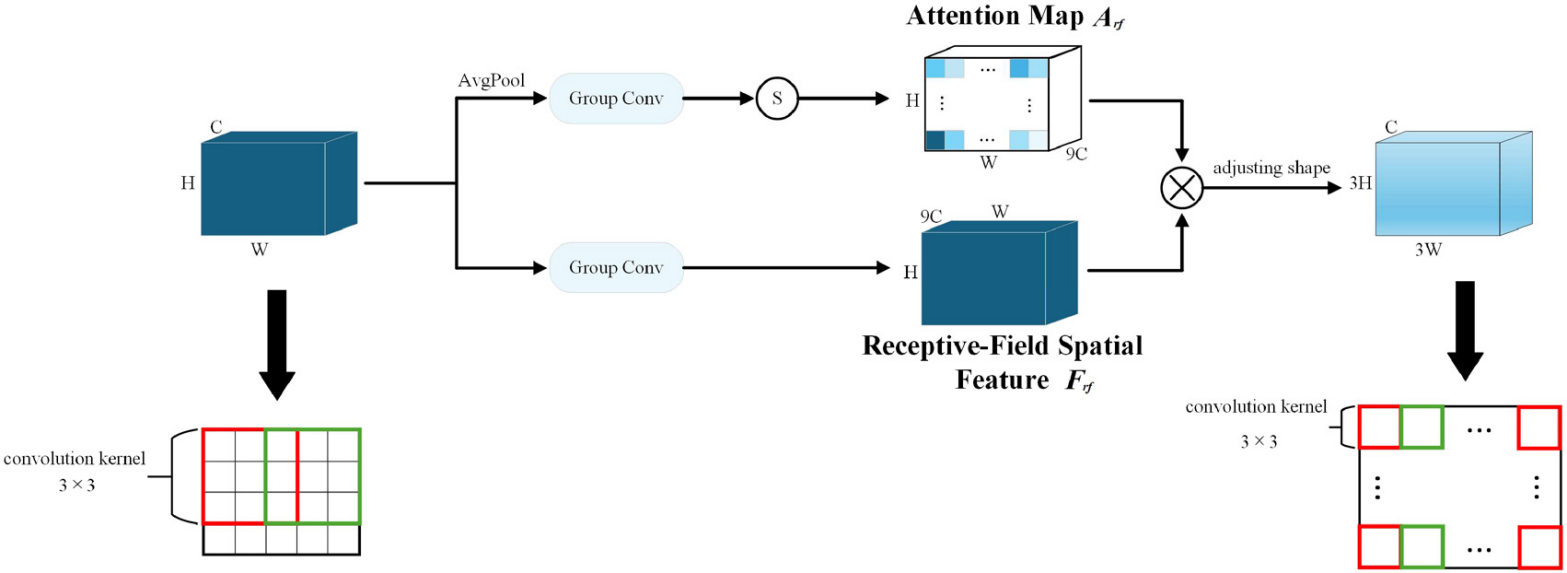
The overall structure of RFAConv. S, Softmax.

The proposal of RFA solves the limitations of the existing spatial attention mechanism and provides an innovative solution for spatial processing. Among them, the Receptive-Field Attention Convolution (RFAConv) (Zhang et al., 2023a) designed based on RFA not only emphasizes the importance of different features within the receptive field slider but also gives priority to the receptive field space features, completely solving the problem of convolution kernel parameter sharing, as shown in **Figure 4**.

In RFA, the entire operation process can be divided into two parts. The first part uses group convolution to extract receptive field spatial features quickly. The second part learns the attention map by interacting with the receptive field feature information to enhance the network’s ability to extract features. However, allowing each receptive field feature to interact will incur a large computational cost. To reduce the computational cost and parameter amount as much as possible, AvgPool is first used to fuse the global information of each receptive field feature, followed by a 1×1 group convolution operation to interact with the information. Finally, the Softmax function obtains the importance of each feature in the receptive field feature. After both parts are completed, the final feature information is obtained by multiplication, as shown in Equation 1.


(1) $$\begin{array}{c} F=Softmax(g^{i\times i}(AvgPool(X)))\times ReLU(Norm(g^{k\times k}(X)))\\ =A_{rf}\times F_{rf} \end{array}$$

where, $A_{rf}$ and $F_{rf}$ represent the attention map and the transformed receptive field space feature map, respectively; $g^{i\times i}$ and $g^{k\times k}$ are group convolutions of size i×i and k×k , respectively; Norm and *X* are normalization and input features, respectively.

The feature map obtained through RFA will not overlap the receptive fields after shape adjustment. Therefore, the learned attention map not only contains all the feature information in each receptive field but does not need to be shared in each receptive field. Finally, a standard convolution with a convolution kernel of k×k and a stride of *k* is used to extract feature information.

**Figure 5:**
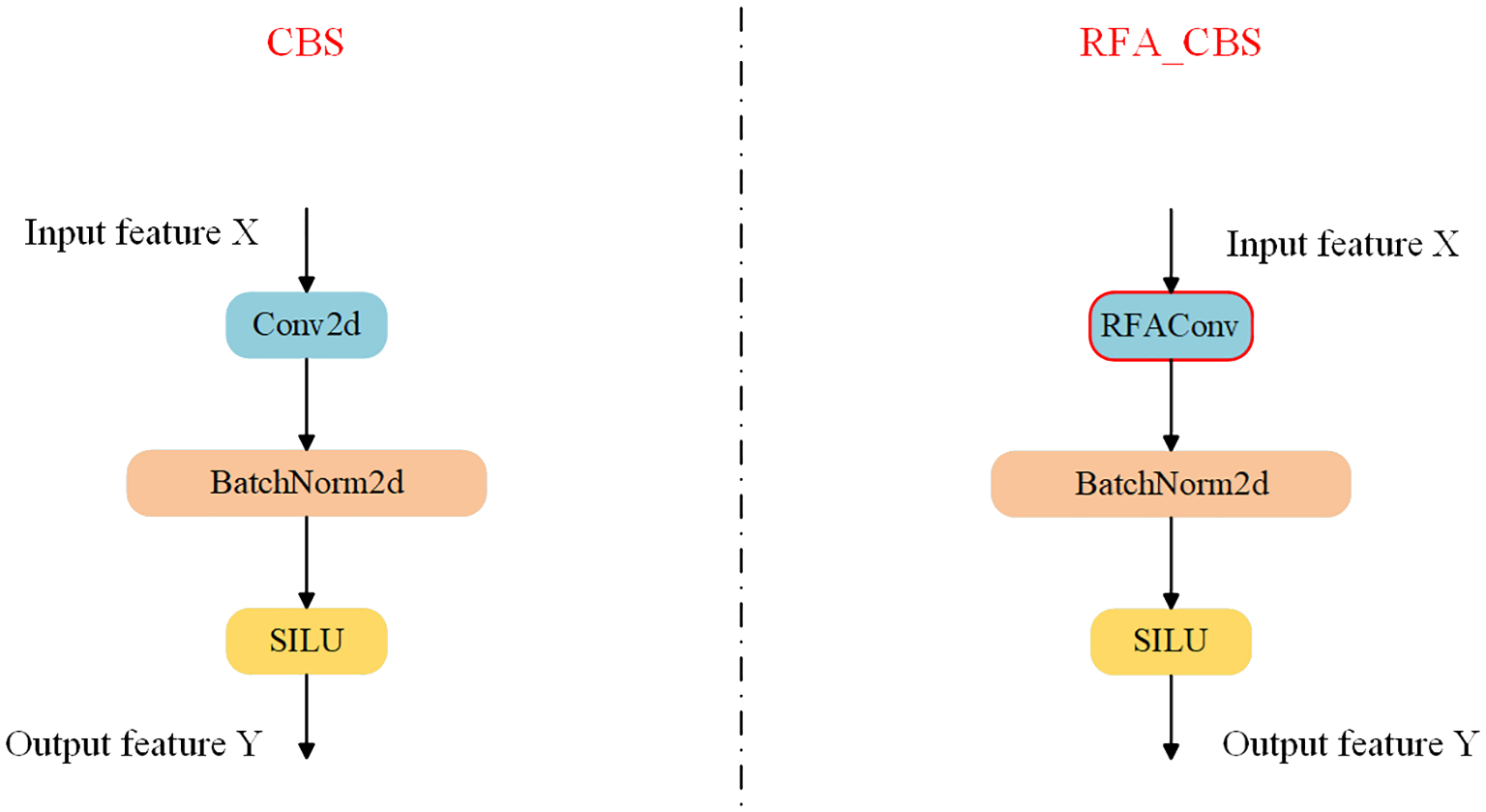
Comparison chart before and after CBS module improvement.

Consequently, in this paper, by replacing the standard convolutional Conv in the CBS module of the backbone network with RFAConv as depicted in **Figure 5**, the feature extraction capability of the backbone network is improved, while the increase in the computational cost and the number of parameters are almost negligible.

### 3.2 Mitigation of neck network feature loss and degradation

**Figure 6:**
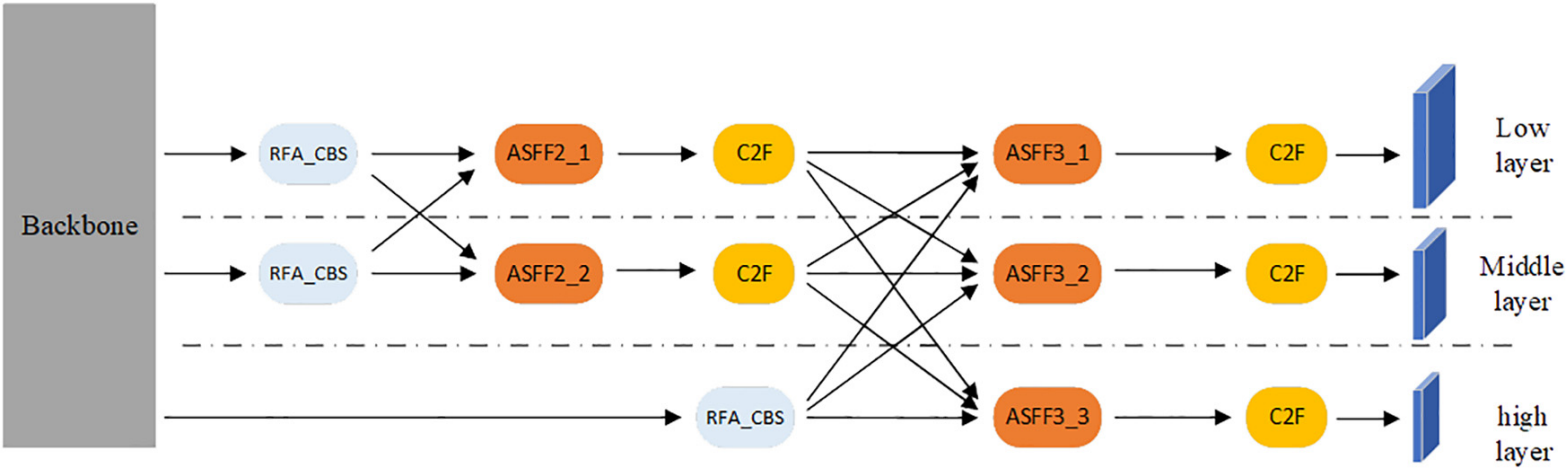
The architecture of AFPN.

In YOLOv8, the main task of the backbone network is feature extraction, but in detection and positioning tasks, the detected objects are multi-scale, and single-scale features cannot be used to detect multi-scale objects. Therefore, YOLOv8 uses the PAFPN structure in the neck network to process the features extracted from the backbone. Initially, the features are fused from top to bottom and then enhanced from bottom to top before generating a multi-scale feature map. Nonetheless, this approach encounters a new issue. In the process of top-down fusion, the high-level feature information may be lost or degraded, while in the bottom-up process, the low-level feature information may be lost or degraded. To address this problem, this paper references the Asymptotic Feature Pyramid Network (AFPN) (Yang et al., 2023) in the neck network, as shown in **Figure 6**, to replace the original PAFPN.

As seen in **Figure 6**, AFPN sequentially fuses the feature information of the bottom, middle, and top layers. This process is carried out gradually, which greatly alleviates the problem of poor feature fusion effect caused by excessive feature differences between non-adjacent layers. For example, feature fusion between the low and middle layers reduces the feature difference between them. Since the middle and high layers are adjacent layers, the feature differences between the low and high layers are also reduced.

The main task of the ASFF module in **Figure 6** is to assign different spatial weights to features at various levels in the multi-level feature fusion process, which enhances the importance of key levels and reduces the impact of conflicting information between different levels. In this article, the ASFF module is divided into two modes, including ASFF2 and ASFF3. Among them, ASSF2_1 and ASSF2_2 denote level 2 feature fusion with two different weights, while ASSF3_1, ASSF3_2, and ASSF3_3 denote level 3 feature fusion with three different weights. Taking level 3 feature fusion as an example, the operation process is as follows:


(2) $${\text{y}}_{ij}^{l}=\alpha_{ij}^{l}\cdot x_{ij}^{1\to l}+\beta_{ij}^{l}\cdot x_{ij}^{2\to l}+\gamma_{ij}^{l}\cdot x_{ij}^{3\to l}$$

where, $x_{ij}^{n\to l}$ represents the feature vector at position (i,j) from level *n* to level *l*; $\alpha_{ij}^{l}$ , $\beta_{ij}^{l}$ , and $\gamma_{ij}^{l}$ are the three spatial weights at level *l*, and the constraint is $\alpha_{ij}^{l}+\beta_{ij}^{l}+\gamma_{ij}^{l}=1$ ; $y_{ij}^{l}$ is the feature obtained after the final fusion.

### 3.3 Improvement of head network

#### 3.3.1 Design of key point detection module

Deep networks extract low-level, mid-level, and high-level features of the input in an end-to-end manner. The richness of feature extraction affects the detection and classification accuracy in the later stages of the network. The network can learn richer features through the number of stacked layers (He et al., 2016), thereby improving detection and classification accuracy. However, as the number of layers (depth) continues to increase, the improvement of network detection and classification accuracy is not absolute. Because each layer of the network also loses part of the feature information while extracting features, the lost features may include some important features, while the extracted features may only be some secondary features and not important features. So, although the number of layers has increased, and the extracted features have become richer, they are likely to be some useless features. Not only will the accuracy not be improved, but the accuracy will be reduced. At the same time, new problems will appear in the network; for example, the gradient may disappear or explode, making the network unable to converge.

**Figure 7:**
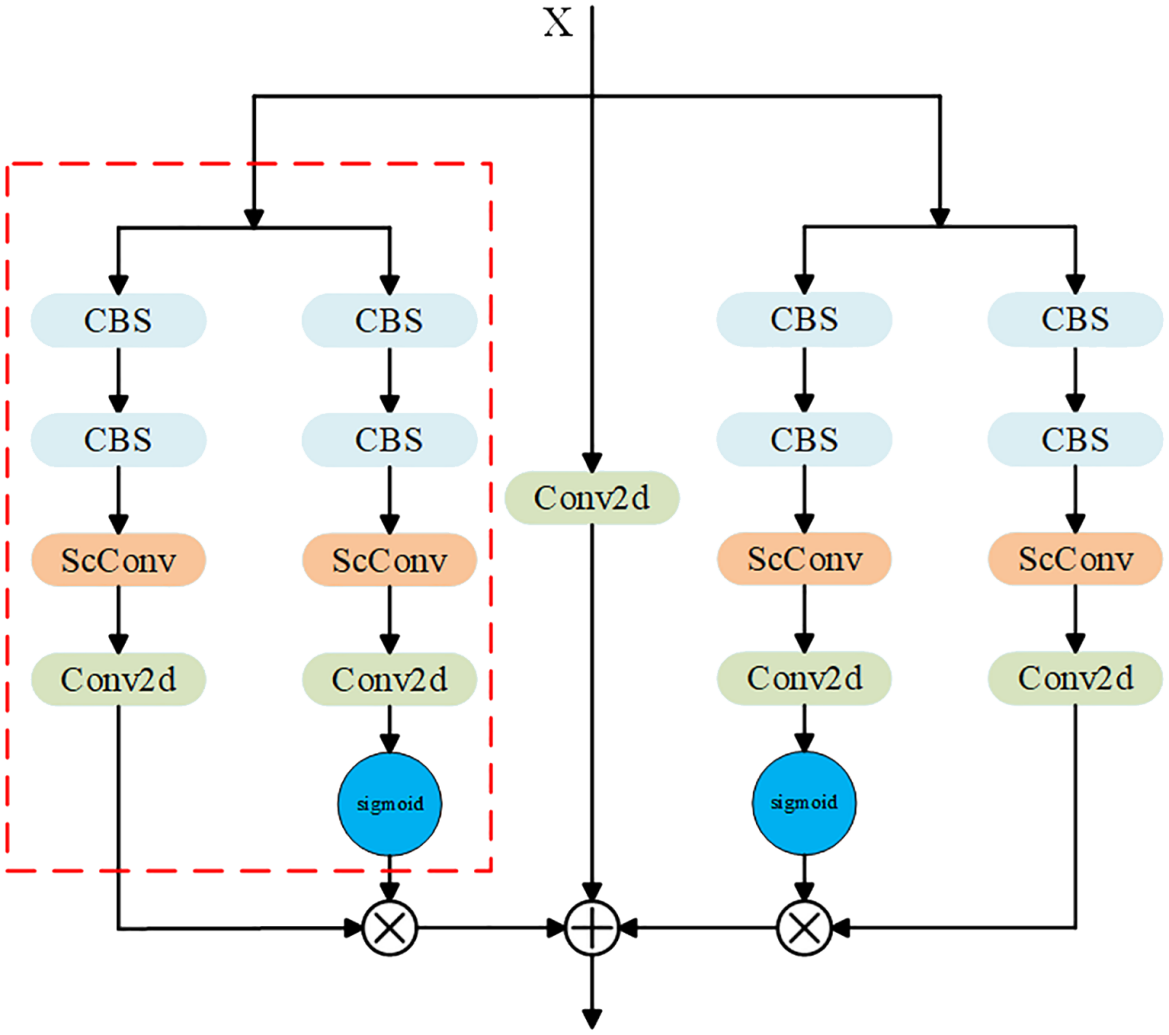
Improved key point detection head.

Therefore, based on ​​the residual network (He et al., 2016), this paper designs a dual-branch key point detection module, as shown in **Figure 7**.

Compared with the standard key point detection head shown in **Figures 3**, **7** consists of four standard points, and two of them are connected in parallel to form a new structure, as shown in the red box in **Figure 7**. In this new structure, a sigmoid function is added to one of the columns to generate a weight value between (0-1). Two identical new structures are connected in parallel, each extracting different features. Then, the importance of the extracted features in the entire module is determined by their respective weight values, w. Finally, the original input X is added to compensate for losing important feature information.

#### 3.3.2 Elimination of redundant features

**Figure 8:**
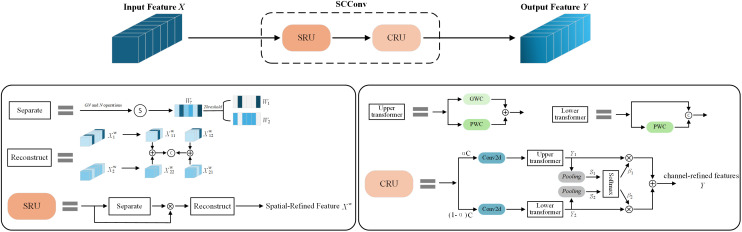
The architecture of SCConv. GN: Group Normalization. N: $w_{i}=\frac{\gamma_{i}}{\sum_{j=0}\gamma_{j}}$ . S, Sigmoid; C, Concatenation.

As the network structure becomes more and more complex, some convolutional layers will extract redundant features, resulting in a huge waste of computing resources. In order to reduce redundant calculations and promote the learning of representative features, this paper adds the Spatial and Channel reconstruction Convolution (SCConv) (Li et al., 2023) to the designed dual-branch key point detection module. SCConv consists of two units: spatial reconstruction unit (SRU) and channel reconstruction unit (CRU), as shown in **Figure 8**. The SRU uses a split-reconstruction method to suppress spatial redundancy, while the CRU employs a split-transform-fusion strategy to reduce channel redundancy.

The SRU consists of two parts: separation operation and reconstruction operation. In the separation operation, the input feature map $X\in {\mathbb{R}}^{N\times C\times H\times W}$ (*N*, *C*, *H*, and *W* are training batch, number of channels, height, and width, respectively) is first standardized to obtain the trainable parameter $\gamma \in {\mathbb{R}}^{C}$ , as shown in Equation 3. Then, γ is normalized to obtain the relevant weight $W_{\gamma }\in {\mathbb{R}}^{C}$ and the weight $W_{\gamma }$ is mapped to (0, 1) using the sigmoid function to indicate the importance of different feature maps, as shown in Equation 4. Finally, the threshold is used for gating to obtain weights $W_{1}$ and $W_{2}$ , while the input feature map *X* is multiplied with it to obtain $X_{1}^{w}$ with rich information and $X_{2}^{w}$ with less information, thus realizing the separation of feature maps with rich information and feature maps with less spatial content, as shown in Equation 5.


(3) $$X_{\text{out}}=GN(X)=\gamma \frac{X-\mu }{\sqrt{\sigma^{2}+\epsilon }}+\beta$$

(4) $$\{\begin{array}{l} W_{\gamma }=\{w_{i}\}=\frac{\gamma_{i}}{\sum_{j=1}^{C}\gamma_{j}},i,j=1,2,\cdot \cdot \cdot ,C,\\ W_{sig}=Sigmoid(W_{\gamma }(GN(X))). \end{array}$$

(5) $$\{\begin{array}{l} W=Gate(Sigmoid(W_{sig})),\\ X_{1}^{w}=W_{1}⊗X,\\ X_{2}^{w}=W_{2}⊗X. \end{array}$$

where, μ and σ are the mean and standard deviation of *X*; ϵ is a small positive number added for division stability; γ and β are trainable affine transformations; ⊗ is an element-wise multiplication.

To maintain the information flow between feature information, the reconstruction operation is used after the separation operation to fully combine the two different information features, so as to enhance the important features and suppress the redundant features in the spatial dimension, and finally obtain the Spatial-Refined Feature Maps $X^{w}$ , as shown in Equation 6.


(6) $$\{\begin{array}{l} X_{11}^{w}⊕X_{22}^{w}=X^{w1},\\ X_{21}^{w}⊕X_{12}^{w}=X^{w2},\\ X^{w1}\cup X^{W2}=X^{w}. \end{array}$$

where, ⊕ is an element-wise summation, and ∪ is the Concatenation operation.

After applying SUR to the intermediate input feature *X*, although the redundant features in the spatial dimension can be suppressed, the redundancy in the channel dimension is still maintained, which is caused by the repeated use of standard convolution with a convolution kernel of k×k . Therefore, in order to eliminate channel redundancy, the channel reconstruction unit (CRU) is introduced to replace the standard convolution.

The CRU consists of three parts: segmentation, transformation, and fusion. First, CRU performs channel segmentation on Spatial-Refined Feature Maps $X^{w}$ , and uses 1×1 convolution to compress the two feature maps obtained after segmentation to improve computational efficiency, and obtains the upper feature $X_{up}$ and the lower feature $X_{low}$ respectively. Then, $X_{up}$ with rich features is sent to the upper transformer, as shown in Equation 7, and $X_{low}$ with a large number of redundant features is sent to the lower transformer, as shown in Equation 8. Finally, the simplified SKNet method is used to adaptively merge the output features $Y_{1}$ and $Y_{2}$ from the upper transformer and the lower transformer, so that the redundancy in the channel dimension is suppressed, and the channel-refined features *Y* is obtained, as shown in Equation 9.


(7) $$Y_{1}=M^{G}X_{up}+M^{P_{1}}X_{up}$$

(8) $$Y_{2}=M^{P_{2}}X_{low}\cup X_{low}$$

(9) $$\{\begin{array}{l} S_{m}=Pooling(Y_{m})=\frac{1}{H\times W}\sum_{i=1}^{H}\sum_{j=1}^{W}Y_{c}(i,j),m=1,2,\\ \beta_{1}=\frac{e^{s_{1}}}{e^{s_{1}}+e^{s_{2}}},\beta_{2}=\frac{e^{s_{2}}}{e^{s_{1}}+e^{s_{2}}},\beta_{1}+\beta_{2}=1,\\ Y=\beta_{1}Y_{1}+\beta_{2}Y_{2}. \end{array}$$

where, $M^{G}\in {\mathbb{R}}^{\frac{\alpha c}{gr}\times k\times k\times c}$ and $M^{P_{1}}\in {\mathbb{R}}^{\frac{\alpha c}{r}\times 1\times 1\times c}$ are the learnable weight matrices of GWC and PWC, respectively; $X_{\text{up}}\in {\mathbb{R}}^{\frac{\alpha c}{r}\times h\times w}$ and $Y_{1}\in {\mathbb{R}}^{c\times h\times w}$ are the upper layer input and output feature maps, respectively; $M^{P_{2}}\in {\mathbb{R}}^{\frac{(1-\alpha )c}{r}\times 1\times 1\times (1-\frac{1-\alpha }{r})c}$ is the learnable matrix of PWC; ∪ is the Concatenation operation; $X_{low}\in {\mathbb{R}}^{\frac{(1-\alpha )c}{r}\times h\times w}$ and $Y_{2}\in {\mathbb{R}}^{c\times h\times w}$ are the lower layer input and output feature maps, respectively.

### 3.4 Model evaluation indicators

This paper evaluates the comprehensive performance of the model through two parts of experiments. The first part of the experiment: Rubber tree tapping surface detection and rubber tapping key point detection accuracy experiments, using Precision (*P*), Recall (*R*), Mean Average Precision (*mAP*), model parameters (Params), Flops, and FPS as evaluation indicators. Among them, *P* and *R* represent the proportion of the number of correctly predicted positive samples to the total number of predicted positive samples and the proportion of the number of correctly predicted positive samples to all positive samples, respectively; *mAP* is the average area under the P-R curve of all categories, which is used to measure the quality of the model in each category, among which *mAP50* is the *mAP* value when the IOU threshold is set to 0.5; FLOPs and FPS respectively show the computing power required for model training and the inference speed of the model (the number of images inferred in 1 second).


(10) $$\{\begin{array}{l} P=\frac{TP}{TP+FP}\\ R=\frac{TP}{TP+FN}\\ mAP=\frac{1}{N}\sum_{i=1}^{N}\int_{0}^{1}P_{i}(R_{i})dR_{i} \end{array}$$

where, *TP*, *FP*, and *FN* represent the number of samples correctly predicted by the model as positive (i.e., the target exists and is predicted to exist), the number of samples incorrectly predicted by the model as positive (i.e., the target does not exist but is predicted to exist), and the number of samples incorrectly predicted by the model as negative (i.e., the target exists but is predicted to not exist); $P_{i}$ , $R_{i}$ and *N* show the precision, recall and number of sample categories, respectively.

The second part of the experiment: Experiment on the positioning accuracy of the starting point and end point of rubber tapping, using *x*-axis offset distance (*xOD*), *y*-axis offset distance (*yOD*), and *xy*-axis offset distance (*xyOD*) as the evaluation indexes. *xOD*, *yOD*, and *xyOD* represent the pixel offset distances between the predicted point and the truth point in the *x*-axis direction, the *y*-axis direction, and the Euclidean direction, respectively. The calculation formulas are as follows:


(11) $$\{\begin{array}{l} xOD=\left|x_{P}-x_{T}\right|\\ yOD=\left|y_{P}-y_{T}\right|\\ xyOD=\sqrt{{\left(x_{P}-x_{T}\right)}^{2}+{\left(y_{P}-y_{T}\right)}^{2}} \end{array}$$

where, $x_{P}$ , $y_{P}$ and $x_{T}$ , $y_{T}$ are the *x*-axis coordinates and *y*-axis coordinates of the predicted point, and the *x*-axis coordinates and *y*-axis coordinates of the truth point, respectively.

## 4 Results and discussion

### 4.1 Ablation experiment

The natural rubber tree tapping surface detection and tapping key point positioning model has been improved in three parts compared to the original YOLOv8n-pose model. Part I A: The convolution RFAConv with receptive field attention mechanism replaces the ordinary convolution in the backbone network CBS module; Part II B: Neck network uses AFPN structure; Part III C: An improved key point detection head is adopted in the Head network. To verify the contribution of each improvement to the entire model, this study conducts an Ablation experiment on the natural rubber tree tapping surface detection and tapping key point positioning model. The results are shown in **Table 2**.

**Table 2:** Comparison results of ablation experiments.

A	B	C	D_P/P_P (%)	D_R/P_R (%)	D_mAP50/P_mAP50 (%)	Params (M)	GFlops (G)	FPS (f/s)
×	×	×	96.3/86.2	97.9/87.6	96.9/84.1	3.08	8.3	200
√	×	×	97.7/87.8	99.1/89.7	97.8/85.6	3.10	8.6	167
×	√	×	98.2/89.1	98.7/89.6	97.6/85.3	3.21	9.6	111
×	×	√	96.4/87.3	97.9/88.4	96.8/86.2	3.16	8.6	143
√	√	√	98.5/88.9	99.2/89.8	98.3/86.4	3.31	10.1	91

^1)^ D_P, D_R, D_mAP50 and P_P, P_R, P_mAP50 denote P, R, and mAP50 for object detection and key point detection, respectively.

The integration of the receptive field attention mechanism has comprehensively improved the P, R, and mAP50 of the object detection and key point detection of the original model. As shown in the experimental results of YOLOv8n-Pose+A, D_P, D_R, and D_mAP50 are improved by 1.4%, 1.2%, and 0.9%, respectively, while P_P, P_R, and P_mAP50 are improved by 1.6%, 2.1%, and 1.5%, respectively, indicating that the feature extraction capability of the backbone network has been enhanced. Meanwhile, the number of parameters and computing power cost has only increased by 0.02M and 0.3G, respectively, further proving that the receptive field attention mechanism has little impact on the size and computing cost of the entire model. After replacing the original PAFPN structure of the Neck network with AFPN, the problem of loss and degradation of bottom-level feature information and top-level feature information has been alleviated. Compared with the original model, the D_P and P_P of the YOLOv8n-Pose+B model are substantially improved by 1.9% and 2.9%, respectively. However, due to the operation of progressive fusion, feature fusion becomes more frequent, which in turn generates more parameters and computing power, increasing by 0.13M and 1.3G, respectively. After designing the original single-branch key point detection head of the Head network into a dual-branch key point detection head and introducing the residual structure, the detection head’s ability to select important features of key points is effectively enhanced. At the same time, the residual structure further compensates for the loss of important features. The P_mAP50 is improved by 2.1% compared with the original model. The combination of RFAConv, AFPN, and enhanced key point detection head showed the best detection performance, with D_mAP50 and P_mAP50 increased by 1.4% and 2.3%, respectively, compared with the original model. Still, it also increased the model complexity, increasing model size and computing power and a slower model inference speed. The biggest impact is the inference speed of the model. Although the FPS dropped to 91, the actual tapping time is 45s, and the tapping speed is about 0.8cm/s. Therefore, the detection speed of 91FPS fully meets the requirements of real-time tapping. The number of model parameters and computing power has only increased slightly, with Params increased to 3.31M and GFlops increased to 10.1G. In the current application of lightweight models in agricultural fields, Sun et al. (2022) proposed a lightweight model with 6.84M Params and 14.7G GFlops for rubber tapping, and Zhang et al. (2023b) proposed a lightweight model with 4.78M Params and 12.3G GFlops for animal recognition. In comparison, YOLOv8n-IRP is much smaller than them in parameters and GFlops, which is very beneficial for deployment on intelligent rubber-tapping machines.

**Figure 9:**
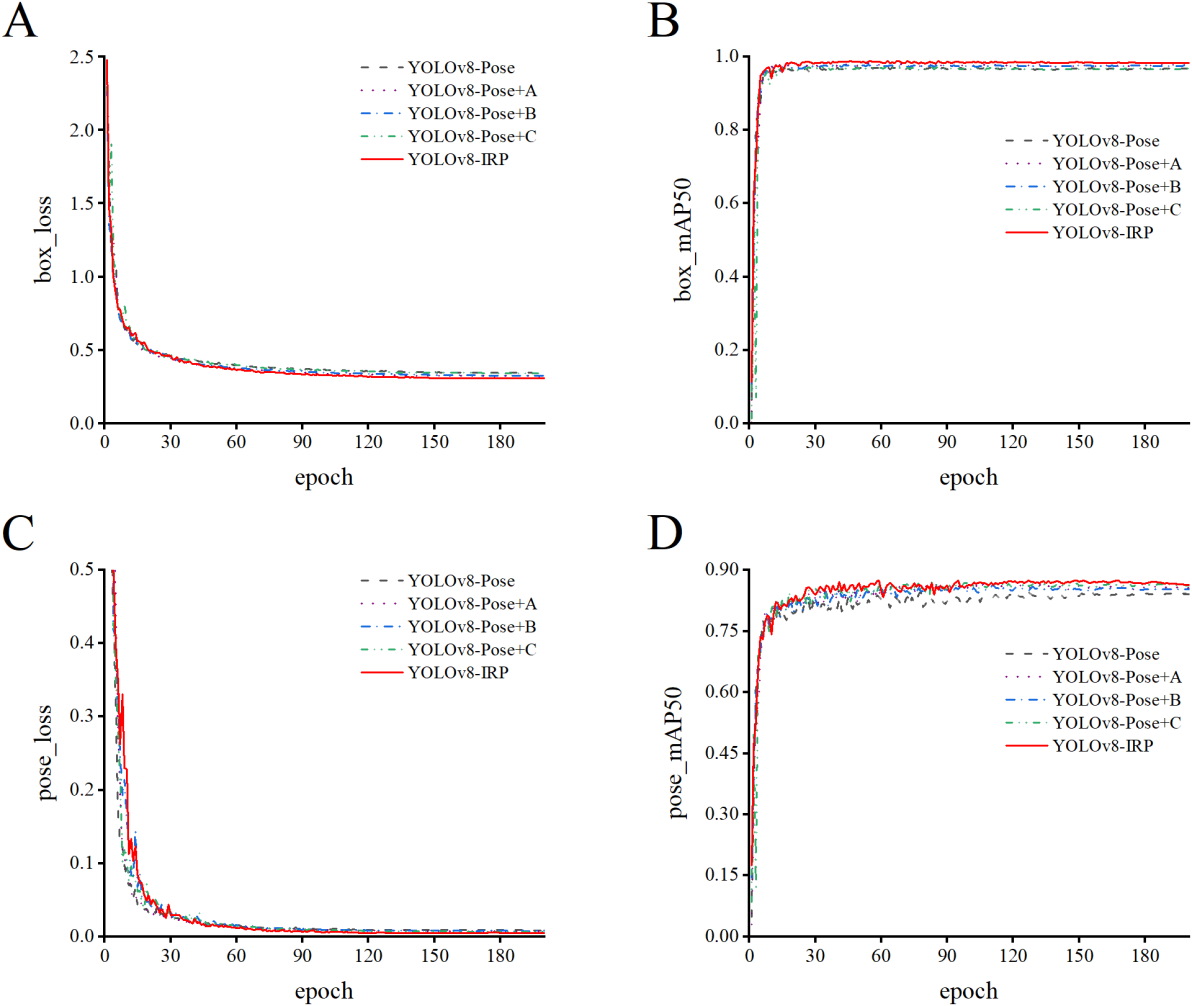
Comparison of loss and mAP50 curves in ablation experiments. **(A, B)** The convergence of the object loss and the D_mAP50. **(C, D)** The convergence of the key point loss and the P_mAP50.

**Figure 9** shows the training results of the model more intuitively. Before 30 epochs, the loss of the object and key points decreases rapidly, while the mAP50 increases rapidly, indicating that the model has a faster convergence rate both before and after the improvement, and does not decrease due to the increase in model complexity. Simultaneously, the improved YOLOv8n-IRP has lower loss and higher mAP50 than the original model. This observation shows that the combination of RFAConv, AFPN, and the improved key point detection head enables the model to have better detection performance.

### 4.2 Comparison of detection performance between different models

To demonstrate the comprehensive performance of the improved YOLOv8n-IRP model in rubber tree tapping surface detection and rubber tapping key point detection, this experiment uses three popular object detection and key point detection algorithms for comparison, as shown in **Table 3**. In **Table 3**, except for Faster_RCNN-RTMPose, the other three algorithms are lightweight models. Among them, the improved lightweight model YOLOv8-IRP has the highest D_P and D_R, P_P, P_R, D_mAP50 and P_mAP50, which are second only to the Faster_RCNN-RTMPose, reaching 98.5%, 88.9%, 99.2%, 89.8%, 98.3% and 86.4%, respectively. The reason why the Faster_RCNN-RTMPose can show high detection accuracy in key point detection is due to the detection mode of RTMPose. The RTMPose is a top-down key point detection algorithm. It first detects the object box and then predicts the key points in the object box by generating a key point heat map. This makes detection accuracy better than lightweight models that simultaneously predict objects and key points through regression. However, it also exposes its shortcomings. It needs to train two models: the object detection model Faster_RCNN and the key point detection model RTMPose, which makes its model size larger and requires higher computing power to train the model. The increase in model size and the cumbersome detection steps also greatly reduce the detection speed. As shown in **Table 3**, the Faster_RCNN-RTMPose has the largest Params and GFlops, reaching 54.42M and 199.5G, respectively, and the smallest FPS, only 13f/s, which is extremely disadvantageous for deployment on mobile devices. On the other hand, the Params and GFlops of the YOLOv8n-IRP have only 3.31M and 10.1G, which are dozens of times smaller than the Faster_RCNN-RTMPose. At the same time, the FPS can reach 91f/s, which is several times faster than the Faster_RCNN-RTMPose, so it is more suitable for deployment on mobile devices.

**Table 3:** Comparison results of detection performance of different network models.

Model	D_P/P_P (%)	D_R/P_R (%)	D_mAP50/P_mAP50 (%)	Params (M)	GFlops (G)	FPS (f/s)
Faster_RCNN-RTMPose	96.5/96.6	99.7/97.9	98.7/93.0	54.42	199.5	13
YOLOv5n-Pose	97.1/86.7	98.2/87.8	96.4/83.6	2.58	7.3	167
YOLOv8n-Pose	96.3/86.2	97.9/87.6	96.9/84.1	3.08	8.3	200
YOLOv8n-IRP	98.5/88.9	99.2/89.8	98.3/86.4	3.31	10.1	91

**Figure 10:**
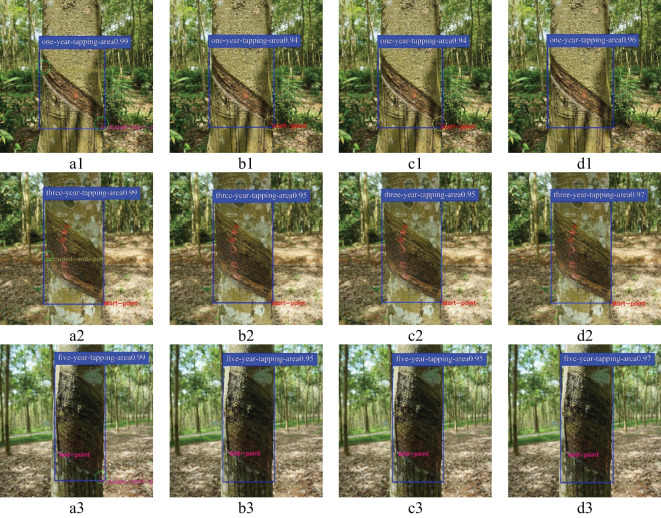
The detection results of different tapping ages trees. Letters **(A–D)** represent the detection results of the Faster_RCNN-RTMPose, YOLOv5n-Pose, YOLOv8n-Pose, and YOLOv8n-IRP, respectively. Numbers 1, 2, and 3 denote rubber trees with one, three, and five year(s) of tapping age.

The rubber forest mainly includes rubber trees with one, three, and five year(s) of tapping age. Therefore, this experiment visualizes the detection results of four models for rubber trees with one, three, and five years of harvesting age, as shown in **Figure 10**. Among them, YOLOv8-IRP achieves more than 96% confidence in the detection of tapping surfaces at one, three, and five year(s), which is 2-3% higher than YOLOv5n-Pose and YOLOv8n-Pose, and it can accurately detect the presence of the starting and end point. Although compared with the Faster_RCNN-RTMPose, it fails to predict the occluded key points (the occluded key points predicted by the Faster_RCNN-RTMPose are shown in the green dotted circles in **Figure 10**), in actual rubber tapping, rubber tapping can only be carried out if the tapping key points are revealed. The occluded starting and ending points have no practical significance for rubber tapping. Therefore, the detection accuracy of the YOLOv8n-IRP meets the requirements of rubber tapping. In addition, The YOLOv5n-Pose and YOLOv8n-Pose have false detection in key point detection, which is mainly manifested in detecting key points from the tapping surface without key points, as shown in the yellow dotted circle in **Figure 10**. This is because the tapping surfaces that expose key points have similar features to those that do not, while the Neck network structure of both YOLOv5n-Pose and YOLOv8n-Pose is PAFPN. The loss or degradation of low-level and high-level features will occur during the feature fusion process. Therefore, it is not possible to distinguish such similar features well enough to make correct predictions. To this end, this paper first uses the RFAConv in the YOLOv8n-IRP to enhance the ability of feature extraction. Then, it uses the AFPN structure to reduce the loss and degradation of low-level and high-level features in the feature fusion process. Finally, the designed dual-branch key point detection head is used to improve the feature screening ability and solve the problem of low prediction accuracy of similar features.

**Figure 11:**
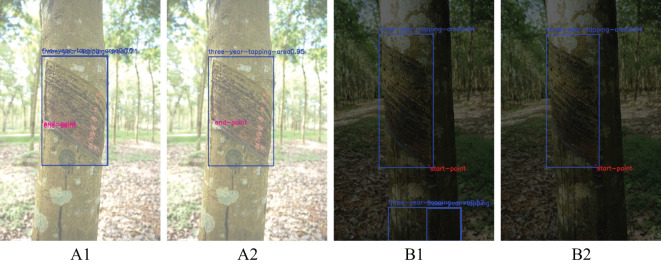
Comparison of detection results under different exposure environments before and after model improvement. Letters **(A, B)** indicate overexposure and underexposure environments, respectively. Numbers 1 and 2 denote the YOLOv8n-Pose and YOLOv8n-IRP models, respectively.

In addition to this, the uncertainty of weather and the shading of light by rubber tree trunk foliage result in variable lighting, which is one of the main challenges for vision applications in rubber forests. Therefore, in order to further demonstrate the usefulness of the improved YOLOv8n-IRP model in rubber forests, this experiment is conducted to test the overexposed, underexposed and normally exposed pictures using four models, respectively, and the detection success rates of the four models in the face of different lighting conditions are counted, as shown in **Table 4**. In **Table 4**, the overexposed, underexposed and normal exposure images used for testing are 200 images, respectively, in which the YOLOv8n-IRP model achieves a detection success rate of 91% in the normal exposure environment, which is more than 5% higher compared to both YOLOv5n-Pose and YOLOv8n-Pose, achieving a higher detection accuracy. For overexposure and underexposure, the detection success rates of the four models have decreased to different degrees, which is caused by 1) the insufficient number of images of complex scenes in the training set and 2) the increased difficulty of extracting important features in complex scenes, which makes the models suffer from the phenomena of misdetection and underdetection. Although the accuracy of YOLOv8n-IRP is reduced by the influence of complex illumination conditions, it still maintains an average detection success rate of 87%, which significantly improves the detection accuracy compared with the original model YOLOv8n-Pose. As shown in **Figure 11**, the duplicate detection and misdetection that originally appeared in overexposure and underexposure are improved, which indicates that YOLOv8n-IRP has a more excellent feature extraction capability and enhanced robustness. While Faster_RCNN-RTMPose has a slightly higher detection accuracy than YOLOv8n-IRP in various exposure scenarios, YOLOv8n-IRP is more suitable to be deployed in mobile devices for intelligent rubber tapping use, considering the detection accuracy, model size, detection speed and the actual situation of rubber tapping.

**Table 4:** Detection results of different models under different lighting conditions.

Model	Light intensity	NSD	NFD	DSR (%)	ADSR (%)
Faster_RCNN-RTMPose	overexposed	169	31	84.5	89.5
	underexposed	180	20	90	
	normal exposure	188	12	94	
YOLOv5n-Pose	overexposed	147	53	73.5	79
	underexposed	160	40	80	
	normal exposure	167	33	83.5	
YOLOv8n-Pose	overexposed	154	46	77	80
	underexposed	157	43	78.5	
	normal exposure	169	31	84.5	
YOLOv8n-IRP	overexposed	168	32	84	87
	underexposed	171	29	85.5	
	normal exposure	182	18	91	

^1)^ NSD, Number of successful detections; NFD, Number of failed detections; DSR, Detection success rate; ADSR, Average detection success rate.

### 4.3 Key point positioning performance comparison experiment

**Figure 12:**
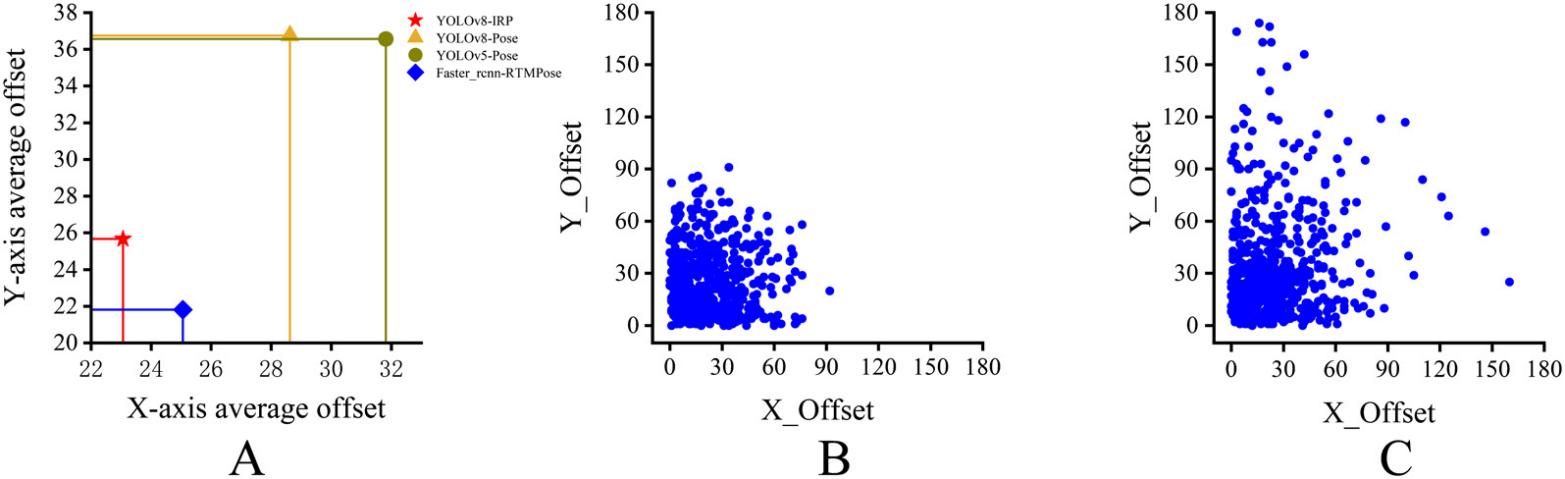
Error distribution scatterplot. **(A)** The average error of the 550 key points predicted by each model in the x-axis and y-axis directions. **(B)** The error distribution of 550 key points predicted by the YOLOv8n-IRP. **(C)** The error distribution of 550 key points predicted by the YOLOv8n-Pose.

To demonstrate the positioning accuracy of the improved YOLOv8n-IRP model at the starting and ending points of rubber tapping, this experiment calculates the *xOD*, *yOD*, and *xyOD* of 550 key points predicted by the four models, and their average values ​​are shown in **Table 5** and **Figure 12**. As can be seen from **Figure 12A**, the average error of the YOLOv8n-IRP on the *x*-axis and *y*-axis is lower than that of the YOLOv8n-Pose and YOLOv5n-Pose, and the accuracy has been significantly improved. It can be seen from **Table 5** that the average offset error of the YOLOv8n-IRP in the x-axis direction is only 23.05 pixels, which is the smallest error among the four models; the average offset error in the y-axis and Euclidean directions is similar to that of the Faster_RCNN-RTMPose and is reduced by more than 10 pixels compared to YOLOv8n-Pose and YOLOv5n-Pose. From **Figures 12B, C**, it can be seen that the stability of the localization error of YOLOv8n-IRP is greatly improved compared with that of YOLOv8n-Pose, in which the maximum error does not exceed 100 pixels, while YOLOv8n-Pose shows an error of close to 180 pixels, further proving that the positioning accuracy is improved after the model improvement. Positioning accuracy and stability affects the regularity of the tapping surface, which in turn affects the efficiency of glue flow. Therefore, the improvement of YOLOv8n-IRP positioning performance has improved the efficiency of glue flow, thereby increasing the latex yield.

**Table 5:** Experimental results of comparing the positioning accuracy of different models.

Model	X-axis average offset(pixel)	Y-axis average offset(pixel)	Average Euclidean distance(pixel)
Faster_RCNN-RTMPose	25.05	21.80	33.18
YOLOv5n-Pose	31.81	36.56	53.53
YOLOv8n-Pose	28.62	36.75	51.41
YOLOv8n-IRP	23.05	25.67	38.45

**Figure 13:**
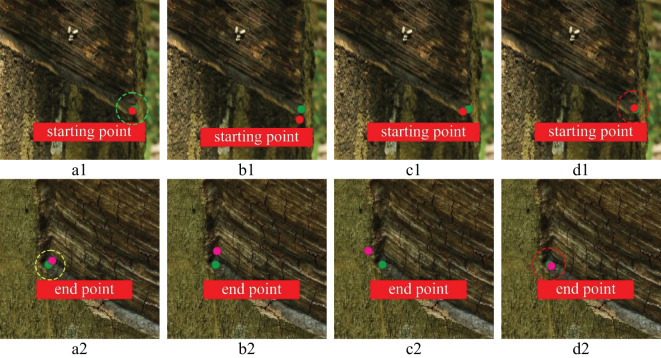
The positioning of key points on the tapping surface with one year of tapping age. Letters **(A–D)** represent the detection results of the Faster_RCNN-RTMPose, YOLOv5n-Pose, YOLOv8n-Pose, and YOLOv8n-IRP, respectively. Numbers 1 and 2 denote tapping surfaces with starting and end points. The red dot is the predicted starting point of tapping. The pink dot is the predicted end point of tapping. The green dot is the key point of truth.

**Figure 14:**
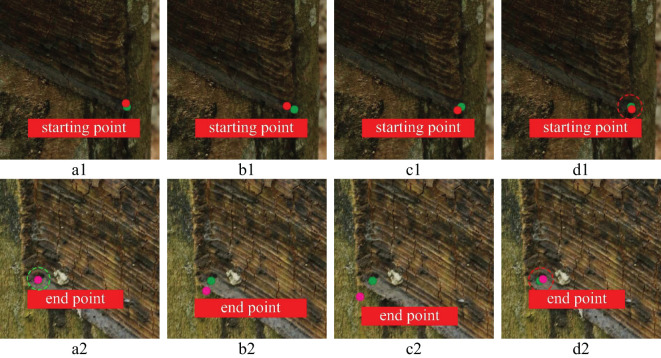
The positioning of key points on the tapping surface with three years of tapping age. Part labels have the same meaning as **Figure 13**.

**Figure 15:**
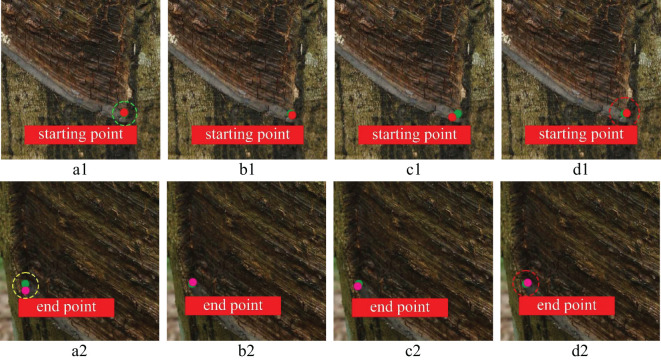
The positioning of key points on the tapping surface with five years of tapping age. Part labels have the same meaning as **Figure 13**.

To further prove the feasibility of key point positioning of the YOLOv8n-IRP model, this experiment visualizes four models’ key point positioning results for rubber trees of different tapping ages, as shown in **Figures 13**–**15**. Among them, the key points predicted by the YOLOv8n-IRP on rubber trees with one, three, and five year(s) of tapping age are close to the truth key points and show high positioning stability, as shown in the red dotted box in **Figures 13**–**15**. However, the positioning deviation of YOLOv8n-Pose and YOLOv5n-Pose are obvious, with large error fluctuations. The Faster_RCNN-RTMPose has the lowest average offset error in the y-axis and Euclidean direction among the four models. Still, it is only a few pixels lower than the improved YOLOv8n-IRP, which is a small improvement for a 4000×6000 pixel photo. Nevertheless, in the visualization experiment, although Faster_RCNN-RTMPose achieves the highest positioning accuracy, as shown by the green dashed box in **Figures 13**–**15**, there were also tapping surfaces with poor positioning, as shown by the yellow dashed box in **Figures 13**–**15**, indicating that the positioning error of Faster_RCNN-RTMPose fluctuates greatly. To sum up, the YOLOv8n-IRP shows better performance in locating key points on the tapping surface, which better meets the rubber tapping requirements.

## 5 Conclusions and future work

In this paper, a rubber tree tapping surface detection and rubber tapping key point localization model is proposed based on the YOLOv8n-Pose. Firstly, the Receptive-field attention mechanism is integrated into the backbone network to solve the problem of sharing common convolutional parameters with larger convolutional kernels, thus improving the feature extraction capability of the backbone network. Secondly, the AFPN is introduced to reduce the loss and degradation of the underlying feature information and the higher-level feature information in feature fusion and enhancement. Finally, a dual-branch key point detection head is designed based on the residual module to improve the feature screening capability. It achieves detecting the tapping surface of different tapping ages and locating the key points of rubber tapping in the complex rubber forest environment, limited storage and computation capacity, with a view to providing a visual guarantee for intelligent rubber-tapping equipment. The main conclusions are as follows:

(1) In the ablation experiment, compared with the YOLOv8n-Pose, the YOLOv8n-IRP has been significantly improved in all aspects of accuracy metrics, in which D_P, P_P, D_R, P_R, D_mAP50, and P_mAP50 have been improved by 2.2%, 2.7%, 1.3%, 2.2%, 1.4%, and 2.3%, respectively. The increase in Params and GFlops and the decrease in FPS are inevitable because the AFPN structure performs feature fusion multiple times in adjacent layers to reduce the loss and degradation of low-level and high-level feature information. Considering the actual tapping speed during rubber tapping, 91f/s is sufficient to meet the rubber tapping requirements. Therefore, it is meaningful to significantly improve the detection accuracy of the rubber tree tapping surface and key points while ensuring that the detection speed meets the rubber tapping requirements.
(2) In the comparative experiment of the detection performance of different models, the D_mAP50 and P_mAP50 of YOLOv8n-IRP reach 98.3% and 86.4%, respectively. The visualization results show that for rubber trees of different tapping ages, the confidence of the tapping surface detection is above 96%, and the unobstructed tapping key points can be detected. The overall detection performance is better than that of YOLOv8n-Pose and YOLOv5n-Pose, which meet the requirements of rubber tapping. Although the Faster_RCNN-RTMPose showed the best detection accuracy, it greatly lost model size and computing power, which is not conducive to deployment in mobile rubber tapping equipment, and the detection speed is not enough to meet the requirements of rubber tapping. Therefore, it is further proved that the YOLOv8n-IRP proposed in this paper is more suitable for intelligent rubber tapping.
(3) In the comparative experiment of positioning performance of different models, the average error between the predicted points of the YOLOv8n-IRP and the corresponding truth points in the Euclidean direction was kept within 40 pixels, which was reduced by 12.96 pixels and 15.08 pixels compared with the YOLOv8n-Pose and YOLOv5n-Pose respectively. The visualization results show that for rubber trees of different tapping ages, the predicted points are close to the truth points, with small fluctuations and stable positioning. The overall positioning performance is similar to the Faster_RCNN-RTMPose, better than the YOLOv8n-Pose and YOLOv5n-Pose, and meets the requirements of rubber tapping.

At present, the method proposed in this paper can accurately detect the tapping surface of natural rubber trees in Danzhou, Hainan. Further research is needed to detect different varieties of rubber trees in other regions, and the positioning accuracy needs to be improved further. In future research, we will collect images of rubber trees of different varieties in different regions, expand the rubber tree data set under different environmental conditions, and study methods to further optimize the network structure and improve the positioning performance. In the entire rubber tapping process, due to the uncertainty of the posture of the rubber trunk, the uncertainty of the attitude of the rubber tree trunk makes it difficult to adjust the end attitude of the robotic arm, so the research on estimating the end attitude of the robotic arm using machine vision is of great significance. Meanwhile, with the integration of different algorithms, the deployment of algorithm models will also bring new challenges, which have higher requirements on the hardware of the rubber-tapping robot, so the research on the lightweight of the model is also of great significance.

## Data Availability

The raw data supporting the conclusions of this article will be made available by the authors, without undue reservation.
